# Supplementary material for: Low Expression of Mitofusin 1 Gene Leads to Mitochondrial Dysfunction and Embryonic Genome Activation Failure in Ovine-Bovine Inter-Species Cloned Embryos
Source: Int J Mol Sci. 2022 Sep 4;23(17):10145. doi: 10.3390/ijms231710145 (PMC9456037; doi:10.3390/ijms231710145)
Supplement: Supplementary file 1 [file ijms-23-10145-s001.zip › ijms-1816808-supplementary.pdf]

Table S1. The development efficiency of IVF, B-B and O-B embryo analysis, respectively

Groups	Reconstructed embryos	2-cell	8-16 cell	Morulae	Blastocyst
IVF	453	362 (79.96) ^{a}	341 (75.45) ^{a}	216 (47.92) ^{a}	144 (31.78) ^{a}
B-B	415	325 (78.36)^{a}	299 (72.05)^{a}	176 (42.41)^{b}	115 (27.71)^{b}
O-B	387	288 (74.46)^{a}	259 (66.96)^{a}	5 (1.38)^{c}	3 (0.78)^{c}

Note: Values with different letters within the same column differ significantly ($P<0.05$).

Table S2. Comparative analysis of the 8-cell and blastocyst development rate of overexpression (OE-Mfn1) or knockdown (shMfn1) of Mfn1 gene in B-B and O-B embryos

Groups		Reconstructed embryos	8-cell	Blastocyst
SCNT	B-B	249	184 (73.93) [a]	70 (28.11)[a]
	B-B OE-Mfn1	238	183 (76.89)[b]	75 (31.51)[b]
	B-B shMfn1	261	150 (57.47)[c]	43 (16.48)[c]
iSCNT	O-B	302	195 (64.56)[a]	2 (0.66)[a]
	O-B OE-Mfn1	292	213 (72.95)[b]	5 (1.71)[b]
	O-B shMfn1	267	135 (50.56)[c]	1 (0.37)[c]

Note: Values with different letters within the same column differ significantly ($P<0.05$).

Table S3. Primer sequences used in this study

Gene	Forward Primer（5'-3'）	Reverse Primer（5'-3'）
Gapdh	GATGGTGAAGGTCGGAGTGAAC	GTCATTGATGGCGACGATGT
Mfn1	ACATCTAGAACGTCTATGGGCATC	CACAAATTGCTGTTTAAAGGCTCTC
Mfn2	CCAACTCCAAGTGTCCGCTTC	TCCAGCTGTCTAGCTCTGTGGTG
Atp1b3	TACTGGAAAGAGCGAAAGCACAGC	GCATCACTACCAAAGCCGACCTG
Mrpl48	AGCGGTTTGAATGCTACGTTTG	CAGTTCTGGCCGAGCTTTGA
Tomm40	AGTTTGTGAACTGGCAGGTGGA	ACGGTGTGATGCTCTGGAGGTAG
Klf4	AGGGAGACGGAGGAGTTCAATGATC	AGGACGAGGATGAGGCTGATGC
Otx1	TGCCAGAGTCCAGAGTTCAGGTC	TTTCTTTGCTGGGCGGCTCTTG
Kdm5b	TTACCGTCTGCTCCACCGCTAC	GAGTGCCTTCTCGTCCTCAATCATG
Tet1	TGCATATACTCAGGTACAGCCAACA	GCCATCCAGGACATTAGCACA
Tfap2c	GATGACCAGCACCTACTTCTG	CACCAACTCCTTCTGACACG
Pou5f1	AAATTAGCCACATCGCCCAGCAG	CCCAGCAGCCTCAAAATCCTCAC
Taf1d	TCGGAGTGCTATGGTCGGTA	AGTGGTGTAGTTTCCCTGCG
Sp1	ACGGCAACAATGGCAGTGAGTC	TGCTGGTTCTGAAGGTTGGAAGTG
Nfya	ACGCAGACACAGCAGCAGATTG	ACTGGCTGATAGACGATGGTTTGC
Nfyb	GCCATGTCCACCTTAGGCTTCG	CTGCTCCACCAATTCCTTTCTCTCC
Dppa4	TCAGTGGATCCTGCCCAGAGTA	TTGCTGTGATCCCTGCTTTGTAA
Nanog	TAAGCACAGGGGGCAAAAGT	ATGGCTAAAAGGGGTGGAGG
